# Measures of physiological stress: a transparent or opaque window into the status, management and conservation of species?

**DOI:** 10.1093/conphys/cou023

**Published:** 2014-06-27

**Authors:** Ben Dantzer, Quinn E. Fletcher, Rudy Boonstra, Michael J. Sheriff

**Affiliations:** 1 Department of Zoology, University of Cambridge, Downing Street, Cambridge CB2 3EJ, UK; 2 Department of Psychology, University of Michigan, Ann Arbor, MI 48109, USA; 3 Département de biologie, chimie et geographie, Université du Québec á Rimouski, Rimouski, QC, Canada G5L 3A1; 4 Centre for the Neurobiology of Stress, University of Toronto Scarborough, Toronto, ON, Canada M1C 1A4; 5 Department of Ecosystem Science and Management, Pennsylvania State University, University Park, PA 16801, USA

**Keywords:** Anthropogenic disturbance, biodiversity, conservation physiology, fitness, meta-analysis, stress

## Abstract

Conservation physiology proposes that measures of physiological stress (glucocorticoid levels) can be used to assess the status and future fate of natural populations. Increases in glucocorticoids may reflect a more challenging environment, suggesting that the influence of human activities on free-living animals could be quantified by measuring glucocorticoids. Biomedical studies suggest that chronic increases in glucocorticoids can have detrimental effects on survival and reproduction, which could influence the viability of populations. Here, we discuss the use of measurements of glucocorticoids in conservation physiology. We first provide an overview of the different methods to quantify glucocorticoids and their utility in conservation physiology. We then discuss five questions we think are essential for conservation physiologists to address. We highlight how intrinsic (e.g. sex, reproductive status, age, recent experiences) and ecological factors (e.g. predation, food availability, snowfall) can, by themselves or through their interactions with anthropogenic disturbances, affect the physiological stress response and mask any general patterns about the effects of anthropogenic disturbances on glucocorticoids. Using a meta-analysis, we show that anthropogenic disturbances are consistently associated with increased glucocorticoids regardless of the type of human disturbance. We also show that males may be more sensitive to anthropogenic disturbances than females and that faecal glucocorticoids, but not baseline plasma glucocorticoids, consistently increase in response to anthropogenic disturbances. Finally, we discuss how increases in glucocorticoids in free-living animals can sometimes enhance survival and reproduction. Unfortunately, our literature analysis indicates that this observation has not yet gained traction, and very few studies have shown that increases in glucocorticoid levels resulting from anthropogenic disturbances decrease survival or reproduction. We think that the use of measures of glucocorticoids in conservation physiology has tremendous potential, but there are still a number of methodological concerns, in addition to several crucial questions that should be addressed.

## Abstract (precis)

We discuss the methodological issues associated with measuring stress hormones in wild animals. We discuss five questions that we think should be considered about the use of stress hormone measurements in conservation physiology. We present a meta-analysis showing that human activities consistently increase stress hormone levels across vertebrates.

## Introduction

The recent loss of global biodiversity is striking. Of the species that have been assessed, the International Union for Conservation of Nature (IUCN) classifies 22% of mammal, 14% of bird, 21% of reptile and 31% of amphibian species as extinct or threatened with extinction (IUCN, 2013). To ameliorate these biodiversity losses, we need a clear understanding of our role in it. How have environmental and anthropogenic disturbances impacted animal survival and reproduction (i.e. fitness)? Ideally, potential changes in fitness could be predicted, but this is made particularly difficult because we generally do not have a benchmark of population (e.g. growth rate, size, distribution) or demographic parameters (e.g. survival, reproduction) against which the predicted changes could be assessed. Conservation physiology proposes to fill this gap by: (i) quantifying the physiological responses of individuals to disturbance; (ii) determining how these responses affect behaviour, survival and reproduction; and (iii) understanding whether individual-level responses scale up to impact the viability of populations (Wikelski and Cooke, 2006; Cooke *et al.*, 2013). Essentially, it will provide a mechanistic link between the individual animals and the demographic outcome.

A major focus in conservation physiology is the causes and consequences of physiological stress (Wingfield *et al.*, 1997; Cockrem, 2005; Walker *et al.*, 2005; Wikelski and Cooke, 2006; Busch and Hayward, 2009). Environmental challenges or anthropogenic disturbances can trigger activation of the vertebrate neuroendocrine axis that often results in the release of stress hormones (glucocorticoids). Glucocorticoids are fundamental to how animals integrate, cope with and respond to both predictable and unpredictable perturbations in their environment, and they are closely tied to individual performance and fitness, and perhaps to population dynamics (Boonstra *et al.*, 1998; Moore and Jessop, 2003; Breuner *et al.*, 2008; Bonier *et al.*, 2009; Crespi *et al.*, 2013). Measures of glucocorticoid levels can therefore provide quantitative information about how environmental changes impact individuals (Tarlow and Blumstein, 2007). They may even act as an ‘early warning system’ to portend future population declines (Wikelski and Cooke, 2006; Drake and Griffen, 2010; Sheriff *et al.*, 2011a; Fefferman and Romero, 2013) and could be used as a ‘trigger point’ in conservation monitoring programmes, where threshold levels of glucocorticoids could be set and predetermined management plans implemented if those thresholds are reached (Lindenmayer *et al.*, 2013).

In this review, we argue that a detailed understanding of physiological stress through measurements of glucocorticoids plays a central role in conservation biology and physiology by aiding our understanding of the consequences of anthropogenic disturbances or global climate change. We caution that there are also many limitations to this approach and a great deal of fundamental research remains to be done in conservation physiology, especially using an experimental approach. We urge more critical and thorough assessments and interpretations of measures of physiological stress, such as glucocorticoid levels obtained from free-living animals.

## What is physiological stress in vertebrates?

Stress is an ambiguous word given that ‘stress’ can be used to define both the environmental perturbation (‘stressor’) and the suite of physiological responses to the perturbation (‘stress’) so that the definition becomes somewhat circular (‘stressors cause stress’). Physiological stress is frequently described as the group of adaptive physiological responses (‘general adaptation syndrome’; Seyle, 1936) to an aversive extrinsic stimulus (a ‘stressor’) that helps to restore internal homeostasis (Cannon, 1932) following exposure to the aversive stimulus. However, it is important to remember that the homeostatic set points that the stress response is trying to restore can change seasonally or according to life history stage, and with other intrinsic (e.g. age, body condition, reproductive status) or extrinsic factors (e.g. weather, predation risk). To account for this, McEwen and Wingfield (2003) introduced the concept of allostasis or ‘maintaining constancy through change’ to physiological ecology. Allostasis describes the range of predictive and responsive physiological processes that assist in the maintenance of homeostasis and explicitly recognizes that homeostatic set points are not fixed and that organisms have evolved physiological mechanisms that enable them to maintain some degree of internal constancy in the face of a variable environment (McEwen and Wingfield, 2003).

Here, we define physiological stress as the multidimensional physiological response to predictable and unpredictable environmental stimuli (stressors) that challenge internal stability or homeostasis, though we recognize that these homeostatic set points can fluctuate. This includes physiological stress responses associated with coping with a change in the environment, such as an unpredictable anthropogenic disturbance. It also includes physiological stress responses that occur in anticipation of environmental changes, such as seasonal changes in food availability or temperature, or in preparation for increased energetic requirements, such as during reproduction or migration. Most of the following discussion concerns the most common measure of physiological stress, glucocorticoid levels.

## Chronic vs. acute stress in conservation physiology

The vertebrate neuroendocrine response is facilitated largely by the hypothalamic–pituitary–adrenal axis (HPA axis; in birds and mammals) or hypothalamic–pituitary–inter-renal axis (HPI axis; in amphibians, fish and reptiles), which are reviewed elsewhere (Sapolsky *et al.*, 2000; Sapolsky, 2002). Environmental challenges that activate these vertebrate neuroendocrine axes can be either acute or chronic stressors. Acute stressors, such as a severe storm or pursuit by a predator, can elevate glucocorticoid levels in the blood and cause a range of physiological and behavioural changes that facilitate coping with the environmental challenge (Wingfield *et al.*, 1998). Although temporary increases in glucocorticoids during reproduction could theoretically threaten the viability of populations by inhibiting reproduction (e.g. foregoing reproduction entirely, causing abortions or brood abandonment; Young *et al.*, 2006; Ouyang *et al.*, 2012), conservation physiologists are, for the most part, interested in sustained or chronic increases in physiological stress. These chronic increases in glucocorticoid levels are thought to inhibit reproduction and may also reduce survivorship (Sapolsky *et al.*, 2000), though the evidence for this in free-living animals is inconsistent (see sections on Questions 4 and 5 below).

Chronic stress has traditionally been thought to represent a dysregulation of the HPA or HPI axis (Sapolsky *et al.*, 1986) or allostatic (McEwen and Wingfield, 2003) or homeostatic overload (Romero *et al.*, 2009) that is brought on by chronic exposure to unpredictable or uncontrollable environmental challenges. Until recently, most physiological ecologists working on natural populations have accepted this traditional view (Boonstra, 2013). We think that this view is likely to apply to animals exposed to anthropogenic disturbances and, thus, it provides insight into what conservation physiologists should look for. The classic symptoms of chronically stressed individuals are as follows: (i) higher baseline plasma glucocorticoid levels; (ii) increased acute increases in plasma glucocorticoids following an environmental challenge; and (iii) an increased amount of time taken to return plasma glucocorticoid levels back to baseline (Sapolsky *et al.*, 1986, 2000; Sapolsky, 1999; Dickens and Romero, 2013). This dysregulation of the HPA or HPI axis is primarily caused by a loss of negative feedback, in which chronic stress decreases the number of glucocorticoid receptors in key regulatory parts of the brain, such as the hippocampus and hypothalamus (Sapolsky *et al.*, 1984; Romero, 2004; Dickens *et al.*, 2009). Chronically stressed individuals therefore tend to experience a larger cumulative exposure to glucocorticoids, which is thought to induce a series of pathological effects (Sapolsky *et al.*, 1986; Sapolsky, 2005; McEwen and Wingfield, 2010), though again this is mostly restricted to studies in humans or laboratory animals (see sections on Questions 4 and 5 below).

## How to measure chronic stress in free-living animals

Conservation physiology is predicated on the assumption that biomarkers of physiological stress can be used to determine reliably whether an environmental challenge (ecological or anthropogenic) induces chronic physiological stress. A crucial endeavour in conservation physiology is therefore to develop standardized methods to quantify chronic stress in free-living animals (Tarlow and Blumstein, 2007; Sheriff *et al.*, 2011b). Glucocorticoid levels are frequently used to quantify physiological or chronic stress (Sapolsky *et al.*, 2000), and there are a number of sources from which glucocorticoids can be measured in free-living animals, namely from blood (plasma or serum), saliva, faeces, urine, hair or feathers (Sheriff *et al.*, 2011b). Each of these different sources carries with it a number of caveats, which have been extensively reviewed elsewhere (Tarlow and Blumstein, 2007; Sheriff *et al.*, 2011b; Breuner *et al.*, 2013). There are also a number of downstream measures (e.g. glucose, free fatty acids, body mass, telomere length, oxidative stress) that can be used as biomarkers of chronic stress. We briefly discuss on these downstream measures very briefly below because they have recently been reviewed elsewhere (Monaghan *et al.*, 2009; Breuner *et al.*, 2013; Dickens and Romero, 2013; Beaulieu and Costantini, 2014). Here, we focus specifically on the potential value of these methods to quantify glucocorticoid levels for conservation physiologists.

### Instantaneous measures of stress: plasma, serum or salivary glucocorticoids

Individuals under chronic stress can exhibit elevated baseline blood glucocorticoids, a stronger increase in blood glucocorticoids in response to some environmental challenge or a reduced ability to terminate the stress response (Sapolsky *et al.*, 1986; Sapolsky, 2002; Dickens and Romero, 2013). Baseline glucocorticoids (Bonier *et al.*, 2009), the magnitude of the stress response (Romero and Wikelski, 2001; Wikelski *et al.*, 2002; Blas *et al.*, 2007; Breuner *et al.*, 2008; MacDougall-Shackleton *et al.*, 2009) and the ability to terminate it (Romero and Wikelski, 2010) may also be tied closely to survival in free-living animals.

Baseline glucocorticoid levels can be measured by obtaining blood samples within ∼3 min of initial capture (Romero and Reed, 2005). In fish and perhaps other aquatic species, baseline glucocorticoid levels may also be measured by determining the amount of glucocorticoids or their metabolites that are excreted into holding water (Wysocki *et al.*, 2006; Scott and Ellis, 2007; Scott *et al.*, 2008). However, these measures of glucocorticoids in holding water may actually represent stress-induced glucocorticoid levels because holding water is generally obtained by moving fish from one large aquarium to another (Wong *et al.*, 2008). Salivary glucocorticoid levels obtained from terrestrial vertebrates are also thought to reflect baseline glucocorticoid levels in the blood with a 20 min lag (Kirschbaum and Hellhammer, 1989). The magnitude of the stress response can be assessed by obtaining a series of blood samples at set time points following a standardized restraint stress test (Romero *et al.*, 2008) or an injection of adrenocorticotrophic hormone (Sheriff *et al.*, 2011b). The magnitude of the stress response can also be assessed in fish by measuring the amount of glucocorticoids excreted into holding water after a simulated stressor (Scott and Ellis, 2007; Wong *et al.*, 2008). The ability to terminate the stress response through negative feedback can be assessed by obtaining a blood sample at a set time point following an injection of dexamethasone, which binds to glucocorticoid receptors and should ultimately cause a decrease in the synthesis of glucocorticoids in the adrenal glands (Sapolsky and Altmann, 1991; Boonstra *et al.*, 1998).

In a recent review of all studies purportedly measuring the effects of chronic stress in laboratory and wild animals, Dickens and Romero (2013) found that baseline or stress-induced glucocorticoid levels in blood samples did not change in a consistent manner in response to chronic stress. Baseline or stress-induced plasma glucocorticoid levels sometimes increased, decreased or did not change in response to chronic stress. In contrast, chronic stress consistently decreased the ability to exert negative feedback and terminate the stress response in nearly all studies using dexamethasone injections (*n *= 19; Dickens and Romero, 2013), though there are some clear exceptions from studies of free-living animals under chronic stress (Boonstra *et al.*, 1998; Sheriff *et al.*, 2011a). The ambiguity in the effects of chronic stress on baseline or stress-induced blood glucocorticoid levels cautions that they may not be useful for conservation physiologists. Some of this ambiguity is likely to be due to the difficulty of obtaining baseline blood samples, at least in mammals (within 3 min of capture; Romero and Reed, 2005), and potentially, habituation to the chronic stress methodology itself that is used in laboratory settings (Cyr and Romero, 2009). It could also be due to differences in measures of total (bound and unbound to corticosteroid-binding globulin) vs. free glucocorticoids (unbound to corticosteroid-binding globulin; Breuner *et al.*, 2013). For example, exposure to physiological stress could decrease corticosteroid-binding globulin, thereby increasing free glucocorticoids (the biologically active portion) while not changing total glucocorticoids (Boonstra *et al.*, 2001).

Unfortunately, measures of the magnitude of the stress response or the ability to terminate the stress response are not practical for most species of conservation concern. They require a series of blood samples to be obtained following restraint and injections (dexamethasone or adrenocorticotrophic hormone) to quantify plasma glucocorticoid levels (Sheriff *et al.*, 2011b). The live-capture, restraint and anaesthesia of mammals to obtain these measures reduces the ability to measure baseline plasma glucocorticoid levels that should be obtained within 3 min of initial capture and can also carry significant costs due to capture-related mortality (Jacques *et al.*, 2009). Measures of salivary glucocorticoid levels in terrestrial vertebrates or measures of glucocorticoids or their metabolites in holding water in aquatic vertebrates have not been used as extensively. Both measures could be useful for conservation physiologists (e.g. Wysocki *et al.*, 2006) given that they may reflect baseline levels (at least in the case of salivary glucocorticoids) but do not require blood drawing. However, obtaining both of these types of samples generally still requires capture and restraint, and so their usefulness in conservation physiology may therefore be limited.

### Integrated measures of glucocorticoids: faeces, urine, feathers and hair

Individuals experiencing chronic stress are thought to experience a higher cumulative exposure to glucocorticoids (Sapolsky *et al.*, 1986; Sapolsky, 2005). Integrated measures of glucocorticoid levels circulating in the blood, such as those from faeces, urine, feathers, hair or holding water (for aquatic species only), reflect an integrated average of blood glucocorticoids that individuals have secreted, metabolized and excreted over a species-specific duration (Sheriff *et al.*, 2011b). Integrated measures of glucocorticoids may therefore more closely reflect the cumulative exposure of individuals to glucocorticoids rather than point samples obtained from blood or saliva and may be the only practical measures that conservation physiologists can use to quantify chronic stress.

Faecal glucocorticoid metabolite (FGM) levels have been widely used in conservation physiology and represent one of the least invasive measures of physiological stress because they can be obtained without capture in some species. Faecal glucocorticoid metabolites reflect free (unbound to corticosteroid-binding globulin) plasma glucocorticoid levels (Sheriff *et al.*, 2010) that an individual has experienced over a species-specific amount of time (Palme *et al.*, 2005; Sheriff *et al.*, 2011b). Urinary glucocorticoid levels may be useful in some contexts but are, in general, extremely difficult to collect from free-living species except perhaps arboreal primate species (but see Creel, 2001 for examples).

The use of hair and feathers as integrated measures of glucocorticoids is still in its infancy, and there are a number of methodological concerns associated with these methods that need to be resolved (Sheriff *et al.*, 2011b; Keckeis *et al.*, 2012; Meyer and Novak, 2012). For example, glucocorticoid levels in hair may not be representative of circulating levels of plasma glucocorticoids (Keckeis *et al.*, 2012), and hair sampled from different parts of the body might have different glucocorticoid levels (Ashley *et al.*, 2011; Terwissen *et al.*, 2013).

In support of their usefulness in measuring chronic stress, Dickens and Romero (2013) found that integrated measures of glucocorticoid levels, such as FGM levels, were significantly higher in response to chronic stress. Although one study in captive birds exposed to chronic stress shows that FGM levels decrease instead of increase (Cyr and Romero, 2008), this seems to be an exception. Of all the different methods available, integrated measures of glucocorticoids, especially FGM levels, appear to be the most reliable indicator of chronic stress and are also perhaps the most practical and least invasive (Sheriff *et al.*, 2011b). Of course, with any measure of physiological stress, they need to be compared against baseline data, such as those before the environmental or anthropogenic disturbance, and not necessarily compared with measures of physiological stress from another population (see section on Question 1 below).

### Downstream measures of chronic stress

Although our focus is on measures of glucocorticoids, there is considerable unexplained variation in the relationship between measures of glucocorticoids and life-history traits (see section on Question 4 below). This may require a broader analysis of physiological stress, such as by measuring the downstream effects of chronic stress (Breuner *et al.*, 2013). Here, we discuss a limited number of metrics that could be useful for conservation physiologists that are reviewed elsewhere (Monaghan *et al.*, 2009; Breuner *et al.*, 2013; Dickens and Romero, 2013).

Glucose levels are predicted to increase in conditions of chronic stress (Fujiwara *et al.*, 1996), which has been observed in some studies in free-living animals (Boonstra *et al.*, 1998; Ruiz *et al.*, 2002; Clinchy *et al.*, 2004; Sheriff *et al.*, 2011a). Free fatty acids and haematocrit levels in free-living animals have been observed to decrease in environments where individuals were presumed to be under chronic stress (Hellgren *et al.*, 1993; Boonstra *et al.*, 1998; Clinchy *et al.*, 2004; Sheriff *et al.*, 2011a). Immune responses are predicted to decline under chronic stress (Sapolsky *et al.*, 2000; Dhabhar, 2009; Martin, 2009), and this has been observed in a number of studies in free-living animals that have used relatively simple measures of immune function, such as counts of leukocytes and lymphocytes (Baker *et al.*, 1998; Boonstra *et al.*, 1998; Hanssen *et al.*, 2003; Clinchy *et al.*, 2004; Davis, 2005; Lobato *et al.*, 2005; Travers *et al.*, 2010; Sheriff *et al.*, 2011a). Chronic stress is also thought to inhibit reproduction (Rivier and Rivest, 1991; Sapolsky *et al.*, 2000; Kirby *et al.*, 2009), and levels of specific reproductive hormones have been observed to decrease in free-living animals thought to be under chronic stress (testosterone: Sapolsky, 1985; Boonstra *et al.*, 1998; progesterone: Hackländer *et al.*, 2003; and prolactin: Delehanty *et al.*, 1997). Measures of oxidative stress (Monaghan *et al.*, 2009; Beaulieu and Costantini, 2014) or telomere shortening rates (Nussey *et al.*, 2014) potentially caused by increased oxidative damage (von Zglinicki, 2002; Monaghan and Haussmann, 2006; Haussmann *et al.*, 2012) or increased physiological stress (Costantini *et al.*, 2011; Haussmann *et al.*, 2012; Herborn *et al.*, 2014) may be useful downstream measures of chronic stress, but they have yet to be used in a conservation context (but see Beaulieu and Costantini, 2014). A drawback for conservation studies is that most of these downstream metrics of chronic stress necessitate the use of blood sampling, with the exception of some reproductive hormone metabolites that can be measured in faeces (Sheriff *et al.*, 2011b).

Body mass is also predicted to decline under chronic stress, and interestingly, it is a consistent biomarker of chronic stress (Dickens and Romero, 2013). Unfortunately, the measurement of body mass in free-living animals typically requires capture and restraint or ingenious ways of training animals to provide such body mass data (e.g. habituation and training individuals to go on to laboratory balances for a food reward).

As a result of these practical drawbacks of downstream measures of chronic stress and because they simply have not been used as frequently, we narrow our discussion to measures of glucocorticoids or the metabolites of glucocorticoids in plasma/serum, faeces, feathers and hair.

## Methodological limitations of measuring chronic stress in wild animals

One of the key considerations in using any method in conservation must be the degree of invasiveness that is possible or desirable (Cooke and O'Connor, 2010). Measures of chronic stress vary in their degree of invasiveness (Sheriff *et al.*, 2011b). However, even with use of the relatively non-invasive methods the application of stress hormones in conservation physiology remains imperfect because of several attributes that we review briefly below.

### Species specificity

All methods to measure chronic stress require an appropriate validation for each species (Sheriff *et al.*, 2011b). For example, many studies have validated the use of enzyme immunoassays to measure FGM levels in ground and tree squirrels (Bosson *et al.*, 2009, 2013; Dantzer *et al.*, 2010; Sheriff *et al.*, 2012). Even in these relatively closely related species, an appropriate and thorough validation must occur (Buchanan and Goldsmith, 2004; Touma and Palme, 2005; Sheriff *et al.*, 2011b). For rare or endangered species where it may not be feasible to obtain the samples required for a rigorous validation, it is appropriate to validate methods with captive or zoo animals of the same species.

### Daily and seasonal changes

Glucocorticoids fluctuate on a normal circadian rhythm, with peaks in blood occurring immediately prior to daily activity and a continuous slow decline throughout the day (Romero, 2002; Sapolsky, 2002). These changes in glucocorticoid levels can cause changes in all other measures of glucocorticoids or their metabolites (in faeces, urine and saliva), with the possible exception of hair and feathers. Glucocorticoids can also fluctuate seasonally, often with peaks occurring during the breeding season (Harper and Austad, 2001; Sheriff *et al.*, 2012). Thus, the potential for daily and seasonal changes in glucocorticoids needs to be avoided by collecting samples at the same time of day or season, or their effects need to be incorporated into the interpretation of results.

### Sex, reproductive status and age

The sex, reproductive status and age of an animal all have the ability to influence adrenal glucocorticoid production (Romero, 2002; see section on Question 1 below). For example, Dantzer *et al.* (2010) found that reproductive condition significantly affected FGM levels in female North American red squirrels (*Tamiasciurus hudsonicus*), with pregnant females having the highest glucocorticoid levels and non-breeding females the lowest; however, reproductive condition did not affect FGM levels in males. Thus, it is important to collect samples from individuals of known reproductive status where possible, but also to understand the impact of changes in glucocorticoid levels in order to interpret results appropriately (see further discussion in the section on Question 1 below).

### Faecal glucocorticoid metabolite levels

As a result of the non-invasive nature of the technique and the relative ease of collection, FGM measures have become increasingly used with free-ranging animals and in conservation studies. However, several confounding factors that can limit the utility of FGM measures are sometimes overlooked, though they have been highlighted in detail (Buchanan and Goldsmith, 2004; Millspaugh and Washburn, 2004; Palme, 2005; Sheriff *et al.*, 2011b; Goymann, 2012). Dietary changes and metabolic rate of animals can alter the way in which hormones are metabolized or excreted, making comparisons across seasons or between populations eating different foods difficult (Goymann *et al.*, 2006; Dantzer *et al.*, 2011; Goymann, 2012). Faecal glucocorticoid metabolite levels can also be affected by precipitation, temperature and microbial degradation after defaecation (Washburn and Millspaugh, 2002; Stetz *et al.*, 2013). Faecal glucocorticoid metabolites may not be evenly distributed throughout the faecal mass and, thus, the entire mass should be homogenized prior to subsampling (Millspaugh and Washburn, 2003; Sheriff *et al.*, 2011b). Any preservation treatment other than freezing can alter FGM levels (Palme, 2005; Sheriff *et al.*, 2011b) and, because of differing affinities for FGMs, the antibody used in the immunoassay will alter FGM results (Sheriff *et al.*, 2011b). This last point highlights that it is generally not appropriate to compare absolute FGM values from two different studies unless standardized field and laboratory techniques are used.

## Essential questions for incorporating measures of physiological stress into conservation physiology

Similar to authors of other reviews, we think that measures of physiological stress, such as glucocorticoids, can be a key tool in conservation physiology. However, we emphasize here that there are five essential questions that should be considered by conservation physiologists if they are to use measures of glucocorticoids to manage populations, to mitigate anthropogenic disturbances or even to inform governmental policy (Cooke and O'Connor, 2010). Our aim within this section is to strengthen the field of conservation physiology by challenging some of the common assumptions in conservation physiology and, more generally, in physiological ecology.

### Question 1: how do intrinsic factors modify physiological stress responses to environmental challenges?

**Figure 1:**
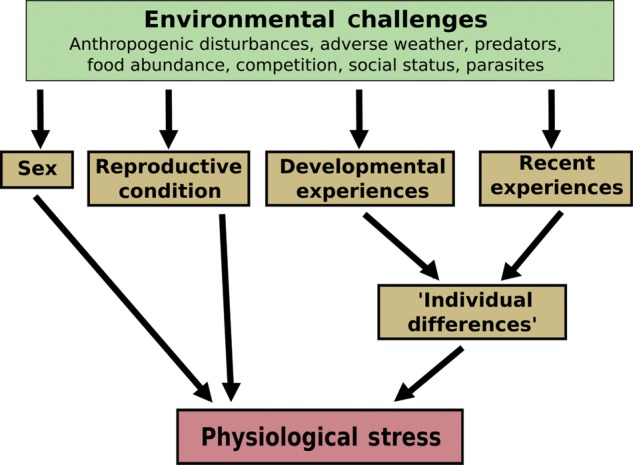
Differences in the intrinsic characteristics of individuals can modify the relationship between environmental challenges (‘stressors’) and measures of physiological stress, such as glucocorticoids. There are many environmental challenges that elicit a physiological stress response, including anthropogenic disturbances and abiotic (weather) or biotic factors (predator or food abundance, competition, social status or parasite load). These anthropogenic and natural environmental challenges can also interact with each other to influence the physiological stress response. The physiological stress response of individuals to these environmental challenges can be modified by the sex of the individual, its reproductive condition (e.g. breeding or non-breeding season, pregnancy, lactation), through ‘individual differences’ that arise due to developmental or recent experiences that either sensitize or desensitize the physiological stress response to environmental challenges.

A variety of intrinsic factors, such as sex, age, reproductive condition, developmental or adult experiences, or interactions between these factors can influence the magnitude of the physiological stress response to environmental challenges or anthropogenic disturbances (Fig. 1). These intrinsic factors may reduce the ability of conservation physiologists to detect physiological stress responses to an anthropogenic disturbance. For example, in free-living northern spotted owls (*Strix occidentalis caurina*), there are sex differences in the effect of logging roads on FGM levels. Male owls but not females living in areas closer to logging roads or timber harvest exhibit higher FGM levels (Wasser *et al.*, 1997). Males also exhibit a much more pronounced rise in FGM levels in response to experimental increases in traffic noise compared with females (Hayward *et al.*, 2011). In contrast, in American kestrels (*Falco sparverius*), only females but not males exhibit higher baseline plasma total corticosterone levels in the presence of anthropogenic disturbances (amount and speed of road traffic, human development: Strasser and Heath, 2013). Thus, not only can there be sex-specific differences in the responsiveness of the HPA or HPI axis to an anthropogenic disturbance, but also the sex that is more responsive may be species specific (see also Ahlering *et al.*, 2013).

In seasonal breeders, changes in reproductive state can influence the magnitude of the physiological stress response to an environmental challenge (Handa *et al.*, 1994). It is still quite rare for studies in conservation physiology to examine how differences in reproductive condition affect the physiological stress response to anthropogenic disturbances. In one exception, in maned wolves (*Chrysocyon brachyurus*) in Brazil, exposure to humans or their activities increased FGM levels in males and non-breeding females but not in reproductively active females (Spercoski *et al.*, 2012). This difference could be because the HPA axis sensitivity of females to environmental challenges tends to be hyporesponsive during pregnancy (Brunton *et al.*, 2008).

A lack of knowledge about the reproductive state of animals from which measures of physiological stress are obtained could reduce the ability of conservation physiologists to make correct inferences about the causes of increased glucocorticoid levels. For example, forest fragmentation has been associated with higher FGM levels in black howler monkeys (*Alouatta pigra*), yet these differences could have also been caused by differences in reproductive state (Martínez-Mota *et al.*, 2007). Female howler monkeys (*n *= 2) in the non-fragmented forest were lactating, whereas those in the fragmented forest (*n *= 2) were not. Given that lactation affects FGM levels (Goymann *et al.*, 2001; Dantzer *et al.*, 2010), this suggests that differences in reproductive status, not fragmentation, may be the cause of these differences in FGM levels in black howler monkeys.

Seasonal changes in reproductive status can also co-occur with changes in anthropogenic disturbances that could lead to false inferences about the causes of physiological stress. For example, exposure to humans through tourism can be associated with increased FGM levels (Barja *et al.*, 2007; Zwijacz-Kozica *et al.*, 2013). However, tourism generally peaks in the spring and summer (Barja *et al.*, 2007; Zwijacz-Kozica *et al.*, 2013), which can coincide with breeding, when FGM levels are elevated (Romero, 2002). As such, tourism pressure and changes in reproductive status co-vary, making it difficult to determine the real cause of changes in glucocorticoid levels without an experimental approach. Future studies employing experimental manipulations of anthropogenic disturbances in both the breeding and non-breeding seasons (Arlettaz *et al.*, 2007; Hayward *et al.*, 2011; Blickley *et al.*, 2012a; Crino *et al.*, 2013) would help to disentangle the multiple causes of increased glucocorticoid levels.

The physiological stress response to environmental challenges can also change with age. In mammalian species, the HPA axis of altricial offspring tends to be hyporesponsive early in life (Wada, 2008), but as individuals grow older, their HPA axis can become hyper-responsive (Sapolsky *et al.*, 1986; Sapolsky and Altmann, 1991). Given that older individuals often exhibit a more prolonged and exaggerated stress response to an environmental challenge, age could magnify the physiological stress response to environmental changes or anthropogenic disturbances. For example, elk (*Cervus elaphus*) in Yellowstone National Park have higher FGM levels when there is greater snowmobile activity in the area, but only after the effects of age and snow pack depth are controlled for statistically (Creel *et al.*, 2002). Faecal glucocorticoid metabolite levels in elk increase with age (Creel *et al.*, 2002), but it is unknown whether age impacts the physiological stress response to snowmobile activity. Older elk might exhibit a stronger increase in FGM levels in response to snowmobile activity such that the age structure of the population could be an important predictor of the effects of human disturbance on measures of physiological stress in elk or other among-population comparisons.

The presence of inter-individual differences in physiological stress responses may also obscure the ability of conservation physiologists to detect general patterns about the effects of anthropogenic disturbances on measures of glucocorticoids (Romero, 2004; Madliger and Love, 2014). Some individuals may consistently exhibit a greater physiological stress response to an environmental challenge (‘reactive individuals’), whereas others consistently exhibit a more dampened stress response (‘proactive individuals’: Korte *et al.*, 2005; Koolhaas *et al.*, 2010). There are multiple causes of inter-individual variation in HPA axis activity and reactivity, such as differences in developmental or early life experiences (Meaney, 2001; Harris and Seckl, 2011). In adults, distant or recent experiences that elicited a physiological stress response can also sensitize (Romero, 2004) or desensitize (Cyr and Romero, 2009; Lynn *et al.*, 2010) the HPA axis response to the same or a novel environmental challenge in the future (Ellenberg *et al.*, 2006; Walker *et al.*, 2006). There are also a growing number of studies documenting that glucocorticoid levels are repeatable within individuals (e.g. Bosson *et al.*, 2009; Dantzer *et al.*, 2010; Ouyang *et al.*, 2011; Cockrem, 2013; Lendvai *et al.*, 2013). What is perhaps more important than documenting that repeatable individual differences exist, however, is to document whether individuals consistently differ in their physiological stress response to changes in intrinsic characteristics (e.g. age, reproductive condition) and extrinsic characteristics (e.g. food availability, weather patterns, anthropogenic disturbances). For example, plasma glucocorticoid levels increase with age in some humans, but decrease with age in others (Lupien *et al.*, 1995, 1998). This highlights that there is among-individual phenotypic plasticity (Nussey *et al.*, 2007) in the effects of age on glucocorticoid levels. This could mask any general patterns about the overall effects that an environmental challenge or anthropogenic disturbance have on a population (Madliger and Love, 2014). For example, in a population subjected to a new anthropogenic disturbance, reactive individuals might exhibit higher FGM levels, whereas proactive individuals might exhibit lower FGM levels, which could result overall in a non-significant change in FGM levels.

We think that these intrinsic characteristics that influence the magnitude of the physiological stress response to environmental challenges or anthropogenic disturbances (Fig. 1) should be an important consideration for conservation physiologists. If there are differences in the sex ratio, age structure, reproductive condition or proportion of reactive vs. proactive individuals, there may be differences in these measures of glucocorticoids regardless of the presence or absence of the anthropogenic disturbance. Given that anthropogenic disturbances could impose new sources of natural selection on behavioural or endocrine traits (Bonier, 2012; Snell-Rood and Wick, 2013), they could alter these population-level parameters (sex ratio, age structure, etc.) in a non-random fashion. For example, anthropogenic disturbances could select for proactive (or bold) individuals with dampened stress responses to human activities (Atwell *et al.*, 2012). This, in addition to the methodological challenges associated with measuring FGM levels (see ‘*Faecal glucocorticoid metabolite levels*’ section above), makes among-population comparisons using FGM levels problematic, though of course in some cases they are unavoidable.

### Question 2: are anthropogenic disturbances consistently associated with increased glucocorticoid levels?

The foundations of conservation physiology include the implicit assumption that physiological stress responses to an environmental challenge are consistent. Do anthropogenic disturbances consistently and uniformly increase glucocorticoid levels or cause chronic stress? To address this question, we performed a meta-analysis to examine the effect sizes of anthropogenic disturbances on baseline and integrated glucocorticoid levels. We calculated 59 effect sizes of the influence of human disturbance on glucocorticoid levels from 46 studies (Table S1; see supplementary material for detailed methods). We categorized four types of human disturbance: noise or disturbance from machines (*n *= 20); tourism (*n *= 13); urban habitat (*n *= 14); and habitat modification (*n *= 12). Our analysis included effect sizes from four different classes of vertebrates: Aves (*n *= 30); Mammalia (*n *= 20); Amphibia (*n *= 3); and Reptilia (*n *= 6). Glucocorticoids were quantified from faecal (*n *= 27), plasma (*n *= 28), hair (*n *= 2), feather (*n *= 1) and urine (*n *= 1) samples (Table S1). The plasma samples that we included in the analysis were considered to reflect baseline levels because they were obtained from animals prior to the capture-related increase in glucocorticoid levels (i.e. <3 min: Romero and Reed, 2005). We did not include studies that quantified stress-induced plasma glucocorticoid levels. We considered glucocorticoid and glucocorticoid metabolite levels measured in feathers, faeces, hair and urine to represent integrated glucocorticoid levels (see ‘Integrated measures of glucocorticoids: serum, urine, feathers and hair’ section above).

Across all vertebrate taxa addressed here, species exposed to human disturbance had significantly higher glucocorticoid levels (baseline plasma and integrated glucocorticoid levels together) when we pooled all effect size estimates from these 59 different effect size estimates. Positive effect sizes indicate that levels of glucocorticoids were higher in response to human disturbance. The weighted estimate of the effect size was 0.32 (J corrected Hedges's *g*; Gurevitch and Hedges, 2001), and the 95% confidence interval (±0.06) indicated that it was significantly greater than zero. An effect size of this magnitude suggests that human disturbance has between a small (0.2) and medium (0.5) effect on glucocorticoid levels (Cohen, 1969). There was one extremely high effect size (10.49) in our analysis, from a study of long-nosed bandicoots (*Perameles nasuta*), where bandicoots in urban areas had substantially higher faecal glucocorticoid metabolite levels than those in less disturbed areas (Dowle *et al.*, 2013). However, excluding this point from our analysis does not have a large effect on the overall effect size (0.31 ± 0.06), indicating that human disturbance significantly increased glucocorticoid levels regardless of whether this outlier was included or not.

**Figure 2:**
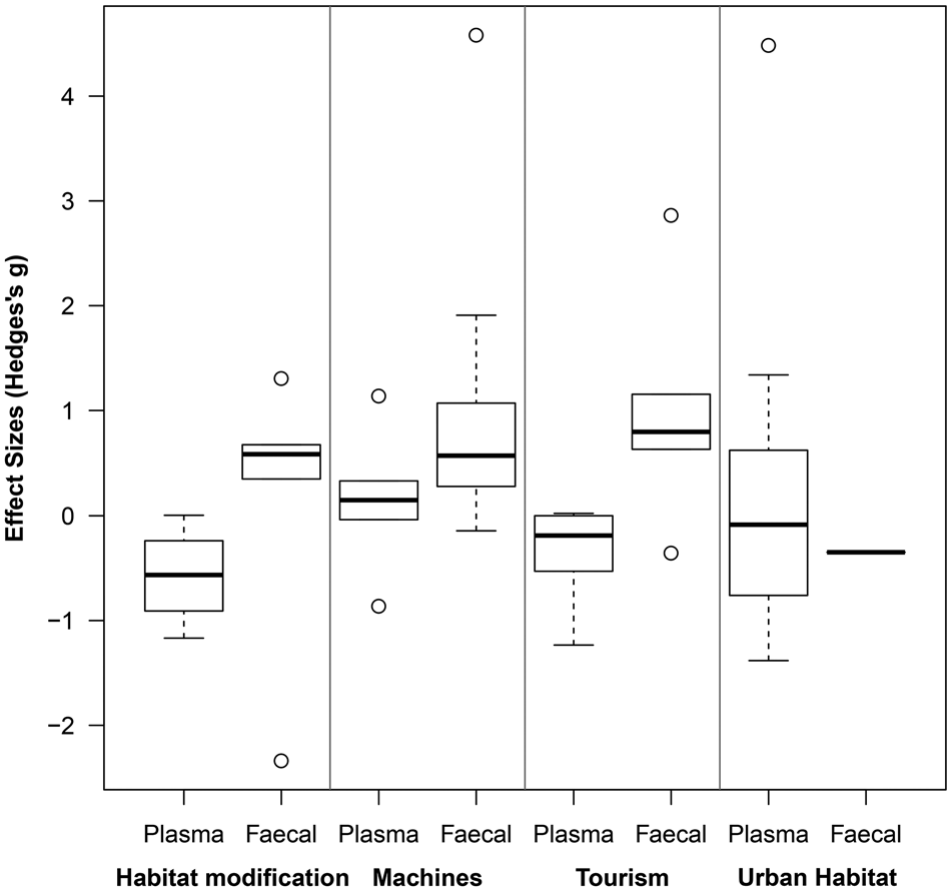
The effect of four different types of human disturbance (habitat modification, machines, tourism and urban habitat) and glucocorticoid sample type (plasma or faecal) on the effect size (Hedges's *g*) associated with human disturbance. Positive effect sizes indicate that glucocorticoid levels were greater in response to human disturbance than in the control conditions. Effect sizes were greater when glucocorticoid levels were calculated using faecal samples compared with blood samples, but there were no differences in effect sizes among the four types of human disturbance (see main text). One extremely high effect size (10.49; Dowle *et al.*, 2013) quantified using faecal samples in the urban habitat category was removed from the figure (see main text). Box (first and third quartiles with median line) and whisker (95% confidence intervals) are shown along with outliers (dots).

We next examined how the four types of human disturbance, glucocorticoid sample type and taxonomy influenced effect sizes in a single linear mixed-effects model (R package metaphor from Viechtbauer, 2010 in R version 3.0.2: R Development Core Team, 2013). In this analysis, we included only glucocorticoid levels quantified in faecal and baseline plasma samples because we did not have a sufficient amount of estimates with the other sample types (hair, feathers and urine; see above). We pooled the samples from amphibians and reptiles into a group called ‘herptiles’ (*n* = 9 studies) and compared them with the samples from mammals and birds. We removed the extremely high effect size from the long-nosed bandicoot (10.49: Dowle *et al.*, 2013) from the analysis because its inclusion changed the interpretation of the effect of disturbance type on glucocorticoid levels (disturbance effect with its inclusion *z* = 2.09, *P* = 0.04). Excluding the estimate from the long-nosed bandicoot, the type of human disturbance did not have a significant effect on the effect sizes (*z* = 1.00, *P* = 0.32; Fig. 2). In this analysis, there was also no significant difference in the effect sizes between herptiles, mammals and birds (*z* = 0.33, *P *= 0.74). Interestingly, this analysis did demonstrate that effect sizes were significantly higher when glucocorticoid levels were quantified in faecal samples compared with when they were quantified using baseline plasma samples (*z* = 2.58, *P* = 0.01; Fig. 2). Specifically, the weighted estimate of the effect size for FGM levels was 0.51 ± 0.08 (95% confidence interval; medium effect: Cohen, 1969), whereas the effect size for plasma sample estimates was not significantly greater than zero (i.e. the 95% confidence interval overlapped zero = 0.09 ± 0.09). This indicates that anthropogenic disturbances were consistently associated with increased FGM levels, but not with increased baseline plasma glucocorticoid levels.

**Figure 3:**
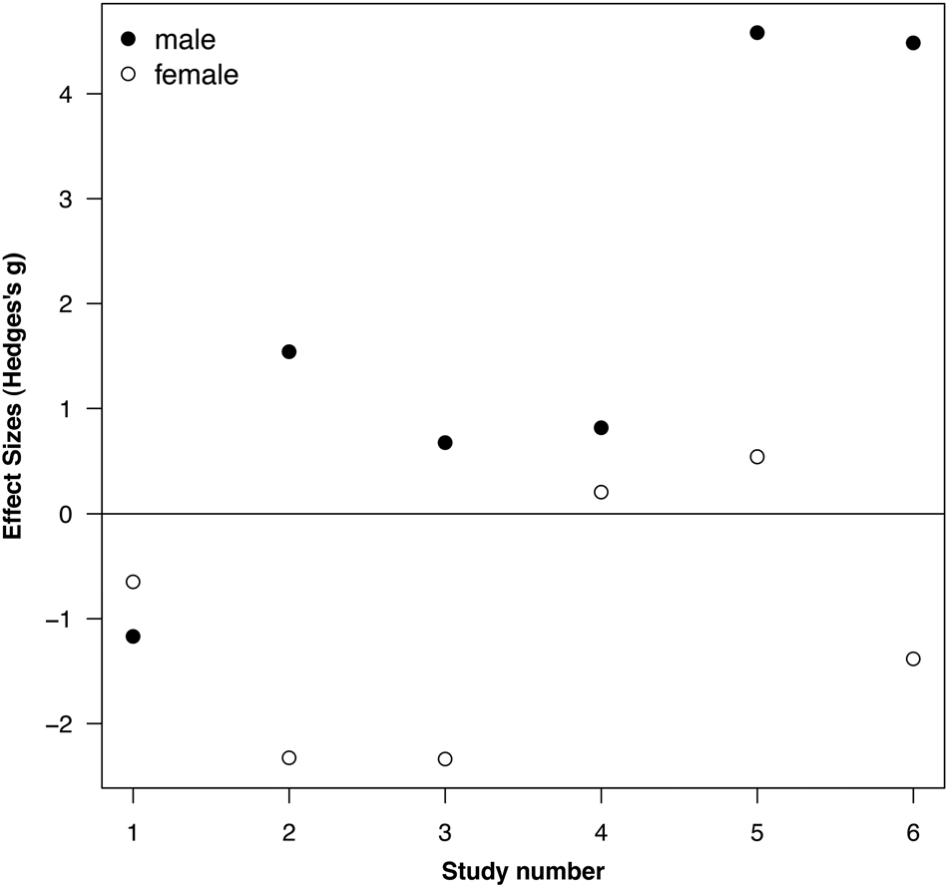
A subset of six studies for which we calculated sex-specific effect sizes because there was a significant human disturbance-by-sex interaction. Positive effect sizes indicate that glucocorticoid levels were greater in response to human disturbance than in the control conditions. Based on this subset of studies, males respond more strongly to human disturbance than females (see discussion in main text). See Supporting information Table S1 for the list of these studies.

In additional analyses, we found that males and females respond differently to human disturbances. We calculated sex-specific effect sizes from six different studies because the original studies found a significant interaction between human disturbance and sex (see Table S1). In five of the six studies, the effect size for males was greater than the effect size for females (Fig. 3). Interestingly, in three out of five of these studies, males had higher glucocorticoid levels in response to human disturbance, whereas females had lower glucocorticoid levels in response to human disturbance (Fig. 3). When these studies were examined using a linear mixed-effects model, the effect size for males was significantly greater than the effect size for females (*z* = 2.60, *P *= 0.009; disturbance and sample type were not included in this model because they were paired). From the remaining studies, we were able to calculate an additional 12 male-specific effect sizes and one additional female-specific effect size. When we included all sex-specific effect sizes in a linear mixed-effects model, once again, the effect sizes for males were greater than the effect sizes for females (*z* = 2.07, *P *= 0.04; sample type was non-significant and was removed from the model). These results suggest that if there is a sex difference, males may respond more strongly to human disturbance than females (e.g. Wasser *et al.*, 1997), though other studies found no significant sex differences in the increase in glucocorticoid levels in response to human disturbances (see Table S1).

There are three fundamental messages from our meta-analysis. First, human disturbance is generally associated with an increase in faecal glucocorticoid metabolite levels, but not in baseline glucocorticoids quantified from plasma samples collected within 3 min of initial capture. Faecal glucocorticoid metabolite levels reflect an integrated measure of glucocorticoids and may more closely reflect the cumulative exposure of individuals to glucocorticoids rather than point samples obtained from baseline blood samples. This indicates that baseline plasma glucocorticoid levels do not consistently increase in response to anthropogenic disturbances. Our results again support our suggestion above that FGM levels may be the most useful measure of glucocorticoids in conservation physiology (see ‘Integrated measures of glucocorticoids: serum, urine, feathers and hair’ section above). Second, all types of human disturbance (habitat modification, machines, tourism and urban habitat; Fig. 2) seem to have a similar effect on glucocorticoid levels. These results are encouraging for conservation physiologists because they suggest that we can assume that the relationship between anthropogenic disturbances and this measure of physiological stress is consistent across species and taxa. Unfortunately, they also suggest that any type of human disturbance causes a substantial increase in glucocorticoid levels. Third, glucocorticoid levels of males may increase to a greater extent in response to human disturbance than glucocorticoid levels of females. As we suggested above (Fig. 1), investigating the effects of anthropogenic disturbances on both sexes is important. The results from our meta-analysis indicate that females may not show increased measures of physiological stress compared with males (but see Strasser and Heath, 2013).

### Question 3: how do intrinsic and ecological factors interact with anthropogenic factors to influence measures of glucocorticoids?

Interactions between the intrinsic and extrinsic factors that we discussed above may also be important because they could sensitize or desensitize the physiological stress response to anthropogenic disturbances (Romero, 2004). For example, FGM levels in elk are influenced by both snowpack depth (ecological factor) and snowmobile activity (anthropogenic factor: Creel *et al.*, 2002). In years of high snowfall, there is greater snowmobile activity and elk have higher FGM levels. However, this is likely not only because of increased snowmobile activity, but also because elk are more vulnerable to predation by wolves (White *et al.*, 2012) and, thus, their HPA axis is sensitized by this higher predation risk.

Anthropogenic disturbances can also desensitize the HPA axis, causing an inappropriate physiological stress response to real ecological challenges. For example, although exposure to chemical pollutants can increase physiological stress (Wikelski *et al.*, 2001, 2002), some of these pollutants (methylsulfonyl polychlorinated biphenyls) can also antagonize the glucocorticoid receptor (Johansson *et al.*, 1998). If pollutants compete with endogenous glucocorticoids to bind to the glucocorticoid receptor, they could desensitize the HPA axis to ecological factors by inhibiting or limiting the normal physiological stress response (Hontela *et al.*, 1992; Boonstra, 2004; Pottinger *et al.*, 2013). Conservation physiologists could wrongly interpret this situation to suggest that the low measures of glucocorticoids in the animals (due to their now compromised HPA axis) were indicative of healthy populations (see also Verboven *et al.*, 2010 for an example where pollutants may sensitize the HPA axis).

The implementation of a conservation action plan can also influence measures of glucocorticoids in unpredictable ways due to interactions between the anthropogenic intervention and ecological causes of physiological stress. For example, elk in North America are provided with supplemental food during the winter (Putman and Staines, 2004). However, elk that were aggregated in the winter in areas with supplemental food had FGM levels that were >31% higher than those in areas without supplemental food and with lower local densities (Forristal *et al.*, 2012), though these differences in FGM levels could also be attributed to diet (Dantzer *et al.*, 2011; Goymann, 2012). If management decisions were based simply on measures of glucocorticoids, the resultant action would be to stop supplemental feeding and reduce population density, which is rarely the goal of conservation physiology.

### Question 4: physiological stress has adverse consequences in humans and laboratory animals, but what about free-living animals?

Short-term increases in glucocorticoids are thought to promote adaptive physiological and behavioural changes, such as those associated with escaping from a predator or coping with a sudden change in weather. Chronic or long-term increases in glucocorticoid levels, however, are widely touted as having detrimental effects, though the evidence comes largely from biomedical studies in humans or laboratory animals (Romero, 2004; Korte *et al.*, 2005; Boonstra, 2013). For example, chronic stress can cause neuronal atrophy in key parts of the brain (Sapolsky, 1996), suppress the immune system (Dhabhar and McEwen, 1997; Korte *et al.*, 2005) and inhibit reproduction (Rivier and Rivest, 1991; Kirby *et al.*, 2009). In contrast, chronic elevations in glucocorticoid levels in free-living animals or those exposed to real ecological challenges can trigger adaptive phenotypic plasticity that enables animals to cope better with that environment (e.g. Dantzer *et al.*, 2013; Middlemis Maher *et al.*, 2013).

Insights from biomedical research about the negative consequences of chronic stress have spurred many ecological studies focused on testing the assumption that fitness is negatively associated with baseline or stress-induced glucocorticoid levels (reviewed by Moore and Jessop, 2003; Breuner *et al.*, 2008; Bonier *et al.*, 2009; Crespi *et al.*, 2013). Specifically, the ‘cort-fitness hypothesis’ predicts that as the number of challenges individuals experience increases, baseline glucocorticoid levels increase and fitness decreases (Bonier *et al.*, 2009). Instead of finding an inverse relationship between baseline plasma glucocorticoid levels and fitness, as would be expected from biomedical studies, Bonier *et al.* (2009) found inconsistent relationships between plasma glucocorticoids and fitness. Of the different studies in vertebrates examined (*n* = 53), 51% found a negative relationship between baseline glucocorticoid levels and fitness, while 30% found a positive relationship.

**Figure 4:**
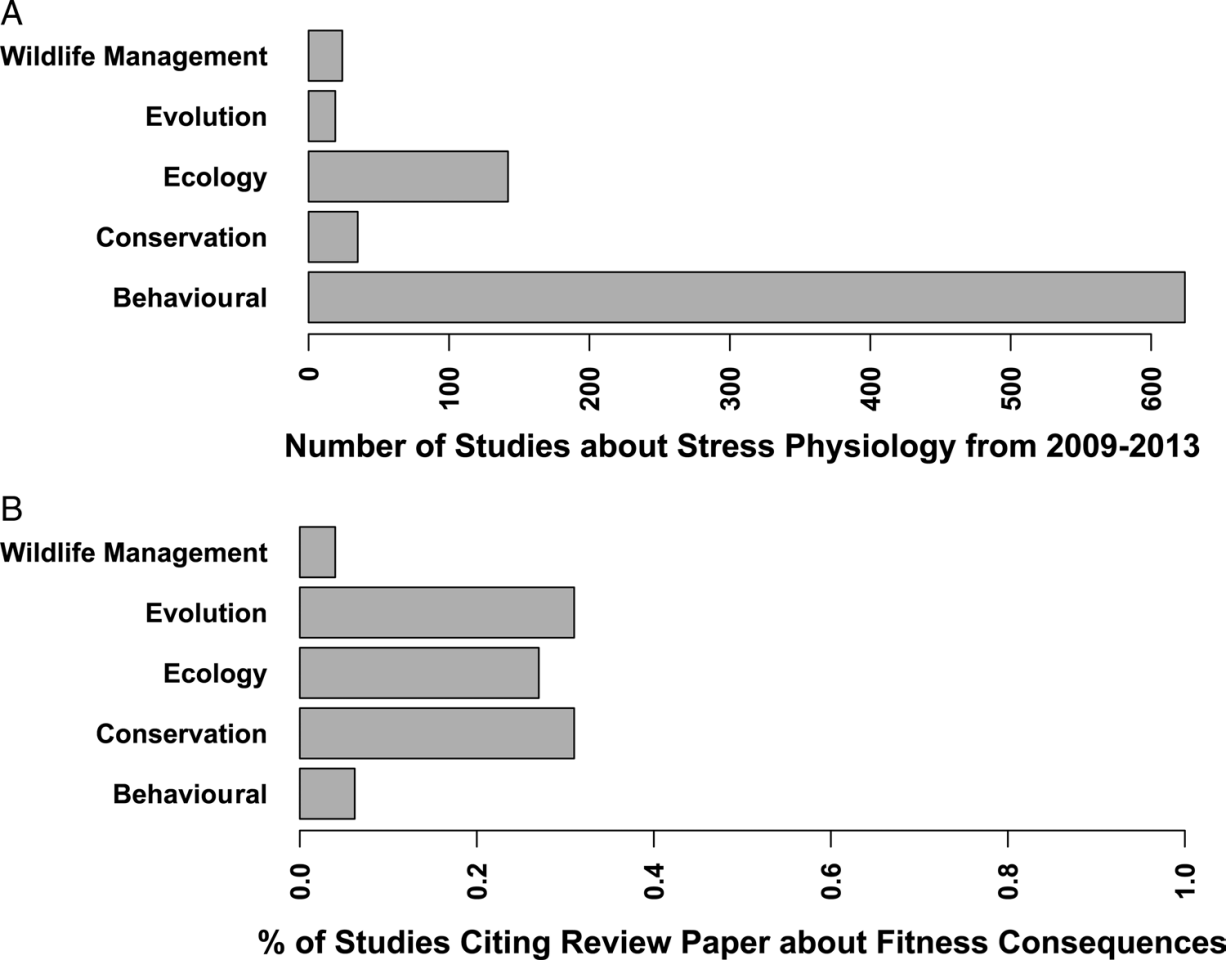
(**A**) The number of studies regarding aspects of physiological stress (using keywords ‘glucocorticoids’, ‘cortisol’ or ‘corticosterone’) in behavioural, conservation, ecological, evolutionary and wildlife management research published from 2009 to 2013. (**B**) The percentage of these studies that cited recent reviews (Breuner *et al.*, 2008; Bonier *et al.*, 2009; Crespi *et al.*, 2013) about the relationships between baseline or stress-induced glucocorticoid levels and survival or reproduction in free-living animals in these different research fields. Search and retrieval of articles was performed through *Web of Science* on 27 December 2013 (complete list available from corresponding author upon request).

The results from Bonier *et al.* (2009) highlight the fact that the relationships between measures of glucocorticoids and fitness in free-living animals, and even in laboratory animals, are inconsistent. We assessed the impact of these reviews (Breuner *et al.*, 2008; Bonier *et al.*, 2009; Crespi *et al.*, 2013) showing that the relationship between measures of physiological stress and fitness in free-living animals is inconsistent by locating all the studies about glucocorticoids (Fig. 4) that were published from 2009 to 2013 (after Bonier *et al.*, 2009 was published) in major journals in the fields of behavioural, conservation, ecological, evolutionary and wildlife management research. There are a growing number of studies about stress physiology published in these areas (Fig. 4A), yet many of the recent studies presumably do not acknowledge the inconsistency of the relationship between glucocorticoids and fitness. From 2009 to 2013, only ∼30% of all the publications about glucocorticoids (Fig. 4B) published in the major journals in conservation, ecology and evolution cite one of the major review papers highlighting these inconsistencies (Breuner *et al.*, 2008; Bonier *et al.*, 2009; Crespi *et al.*, 2013). Only 4% of all of the publications about glucocorticoids published from 2009 to 2013 in the major wildlife management journals cite even one of these review papers.

Although the general assumption derived from biomedical research that increased physiological stress or glucocorticoids causes a reduction in survival or reproduction in free-living animals seems dubious, our basic analysis here shows that this criticism has clearly not taken a hold in physiological ecology, conservation physiology and, especially, not in wildlife management. We acknowledge that this is a coarse analysis and that some of these studies are not specifically focused on measuring the relationship between glucocorticoids and fitness (e.g. those in behavioural journals). However, our emphasis is that most studies in the realm of conservation and wildlife management are likely to discuss how glucocorticoids are closely tied to fitness at some point in the publication, yet they do not cite one of these review papers that indicate how the relationship between fitness and glucocorticoids is not uniformly negative. Unfortunately, many of these studies may be subscribing to the biomedical interpretation of the negative effects of physiological stress and not assessing their data in light of studies of free-living animals.

### Question 5: do anthropogenic disturbances decrease survival and reproduction?

The results from our meta-analysis indicate that anthropogenic disturbances are consistently associated with increased FGM levels across taxa (Fig. 3). However, the fitness consequences of these anthropogenic disturbances or chronic stress caused by the anthropogenic disturbances are rarely investigated. There are a few studies in birds that show that individuals in areas with greater human disturbance (tourism, proximity to road, noise) have higher baseline (Strasser and Heath, 2013) or stress-induced plasma total glucocorticoid (Müllner *et al.*, 2004; Ellenberg *et al.*, 2007) or FGM levels (Hayward *et al.*, 2011) and also exhibit lower survival (Müllner *et al.*, 2004) or breeding success (Ellenberg *et al.*, 2007; Hayward *et al.*, 2011; Strasser and Heath, 2013). Unfortunately, there are a number of alternative explanations for these patterns. In order to ascertain whether reduced breeding success is caused by increased baseline plasma glucocorticoid levels, future studies need to increase glucocorticoid levels experimentally and observe the effects on breeding success. For example, Blickley *et al.* (2012a) experimentally increased FGM levels in lekking male greater sage-grouse (*Centrocercus urophasianus*) using audio playbacks of noises from natural gas drilling and roads, yet the fitness consequences of these experimental increases in glucocorticoids are mostly unknown (but see Blickley *et al.*, 2012b).

Observational studies can also be confounded by the possibility that poor-quality individuals might be forced to breed in suboptimal habitats, such as those near human disturbances. Human disturbance in observational studies is an experimental treatment that it is not applied randomly. Poor-quality individuals might intrinsically show higher baseline plasma glucocorticoid levels and exhibit lower breeding success, but it does not necessarily have to be caused by their proximity to human disturbances. Without an experimental approach, such as randomly applying human disturbances to nests or individuals (e.g. Blickley *et al.*, 2012a, 2012b) distributed within a study area or cross-fostering offspring from a disturbed area to a less disturbed area, it is difficult to determine the direction of causality. A recent study in mountain white-crowned sparrows (*Zonotrochia leucophrys oriantha*) experimentally manipulated the amount of traffic noise to which nestlings were exposed. In contrast to what might be predicted from observational studies, Crino *et al.* (2013) found that nestlings exposed to experimentally increased levels of traffic noise were in better condition (ratio of body mass to tarsal length) and had lower stress-induced plasma total corticosterone levels. Increased exposure to traffic noise also did not affect nestling baseline plasma total corticosterone levels, one measure of immune function, or survival to fledgling.

Future studies employing similar experimental manipulations of anthropogenic disturbances (Arlettaz *et al.*, 2007; Hayward *et al.*, 2011; Blickley *et al.*, 2012a; Crino *et al.*, 2013) should help to illuminate the genuine causes and consequences of increases in glucocorticoid levels due to anthropogenic disturbances. In particular, future studies in the context of conservation physiology should experimentally manipulate glucocorticoid levels in individuals in both disturbed and undisturbed areas using audio playbacks (Blickley *et al.*, 2012a, Dantzer *et al.*, 2013), glucocorticoid provisioning or implants (Silverin, 1986; Dantzer *et al.*, 2013), or by manipulating the activity of glucocorticoid receptors (Landys *et al.*, 2004) or corticotrophin-releasing factor receptors (Xiao *et al.*, 2006). Other experimental manipulations, such as provisioning of supplemental food to some individuals in disturbed habitats but not to others, cross-fostering offspring from disturbed to undisturbed habitats, or altering the intensity and location of tourist pressure will help to determine whether the human disturbance is causing the increases in glucocorticoids and declines in fitness or if these patterns are simply the outcome of poor-quality individuals breeding in areas of high disturbance. We recognize that such manipulations are obviously challenging, but they could be performed in surrogate species to help to understand whether a heightened physiological stress response in areas disturbed by human activity does indeed cause a reduction in survival or breeding success.

Finally, increased glucocorticoid levels in disturbed habitats may reflect coping strategies to deal with those anthropogenic disturbances and may not necessarily cause a reduction in survival and/or reproduction. The allostasis framework (McEwen and Wingfield, 2003) would predict that the stress responses of individuals to environmental challenges are generally adapted to both their life-history strategy and their ecology. For example, bird species that live in areas in which the time to reproduce is short (highly seasonal or cold environments) or stochastic (e.g. arid regions where rainfall is highly variable) exhibit lower acute stress responses (Hau *et al.*, 2010). The dampening of the stress response in these challenging environments might allow individuals to continue to breed despite any environmental challenges they experience (Wingfield *et al.*, 1998; Wingfield and Sapolsky, 2003; Hau *et al.*, 2010). Although the strength of phenotypic selection caused by anthropogenic agents might be increased in comparison to other ecological causes of selection (Hendry *et al.*, 2008; Darimont *et al.*, 2009), adaptation to urban or human-disturbed environments should not be any different. It seems reasonable to conclude that selection will favour endocrine phenotypes that enable individuals to continue to reproduce and survive despite human disturbances (Bonier, 2012). For example, selection in human-disturbed areas might favour individuals with dampened stress responses (Partecke *et al.*, 2006; Atwell *et al.*, 2012). Selection favouring a reduction in the acute stress response could also cause relatively rapid shifts in the HPA or HPI axis of populations in disturbed vs. less disturbed areas (e.g. 12 generations: Atwell *et al.*, 2012). Alternatively, this could mean that individuals in disturbed or urban habitats have increased baseline or stress-induced glucocorticoid levels in comparison to those in less disturbed areas. This increase in cumulative exposure to glucocorticoids does not necessarily need to inhibit reproduction (Wingfield and Sapolsky, 2003) and might not influence the viability of individuals or populations. We think that future studies that link together concepts from evolutionary ecology alongside methods in conservation physiology will be particularly beneficial to addressing how animals adapt to human-disturbed environments through correlated changes in behaviour, physiology and life-history traits.

## Conclusions

*Now I believe that the scattered state of ecology, which is like an active worm that has been chopped into little bits, each admirably brisk, but leading a rather exclusive and lonely existence, and not combining to get anywhere in particular, this disintegrating tendency, in what should be a coherent and united organism, is very largely due to a lack of proper working hypotheses. Charles Elton (1930),* Page 68 in Animal Ecology and Evolution

Almost 100 years ago, the animal ecologist Charles Elton (1930) warned about the lack of coherency in ecology due to a lack of ‘proper working hypotheses’, which is ominous given that there was only one professional publication about animal ecology at that time (*Journal of Animal Ecology*). Importantly, conservation physiology has several working hypotheses, for instance, that measures of glucocorticoids can be used: (i) to document the effects of anthropogenic disturbances on animals; and (ii) to expect or predict future declines in a population or species (Wikelski and Cooke, 2006). However, the underpinnings of these hypotheses are based upon some major assumptions arising from biomedical studies, which seem to be either untested or dubious in free-living animals.

Our review suggests that conservation physiology has not yet gathered all the ‘little bits’ from ecology, evolution and physiology into a single and coherent discipline. This is, of course, not unique; it plagues well-established scientific disciplines and is to be expected from any new scientific discipline. Our review highlights that the physiological stress response to an environmental challenge or anthropogenic disturbance can be modified by a number of intrinsic characteristics that could mask any general patterns. Future studies in conservation physiology should incorporate the effects of sex, reproductive condition and age into their analyses and also be wary of comparisons among populations whose members may differ in these intrinsic characteristics. Our meta-analysis indicates that the presence of anthropogenic disturbances, regardless of their type, consistently and significantly increases glucocorticoid levels in mammals, birds, amphibians and reptiles. However, our further analyses highlight that FGM levels, but not baseline plasma glucocorticoid levels, were higher in response to anthropogenic disturbances and that the effects of anthropogenic disturbances on glucocorticoid levels might be sex specific (Fig. 3). Future studies in conservation physiology must also assess how ecological (food, predators, density) and anthropogenic factors interact with one another to cause or ameliorate physiological stress. Measures of glucocorticoids are used in conservation physiology because they are thought to reflect health or fitness and, therefore, can be used to predict future population declines. This assumption is based upon biomedical studies, and the relationship between fitness and stress physiology is inconsistent in free-living animals and, in some cases, mediates adaptive behavioural or life-history responses to those environmental changes. We think that experimental studies testing the effects of anthropogenic disturbances on measures of physiological stress and their effects on fitness will be particularly valuable in conservation physiology. Finally, there are numerous methodological issues associated with the appropriate measurement of glucocorticoids that need to be considered carefully for all biomarkers of physiological stress but especially for FGM levels.

In this review, we have taken a critical view of the use of measures of glucocorticoids in conservation physiology and wildlife management. We agree with Cooke and O'Connor (2010) that ‘It is also necessary to validate more tools in the “conservation physiology toolbox”, and ensure a thorough understanding of the physiological biomarkers applied to conservation efforts.’ This will undoubtedly require greater coherency in conservation physiology through testing fundamental assumptions and the integration of studies in the other ‘little bits’, including physiology, ecological physiology and evolutionary ecology. We hope that this review exerts stress on the field of conservation physiology to make it a stronger and more robust scientific discipline so that it can be used to slow losses of biodiversity.

## Supplementary material

Supplementary material is available at *Conservation Physiology* online.

Supplementary Data
