# Supplementary material for: Measures of physiological stress: a transparent or opaque window into the status, management and conservation of species?
Source: Conserv Physiol. 2014 Jun 27;2(1):cou023. doi: 10.1093/conphys/cou023 (PMC4732472; doi:10.1093/conphys/cou023)
Supplement: Supplementary Data [file supp_2_1_cou023__index.html]

Measures of physiological stress: a transparent or opaque window into the status, management and conservation of species? — Supplementary Data 

# Measures of physiological stress: a transparent or opaque window into the status, management and conservation of species?

## Supplementary Data

Supplementary Data

**Files in this Data Supplement:**

- Supplementary Data - Doc file
- Supplementary Table 1 - xls file
