# Supplementary material for: Measures of physiological stress: a transparent or opaque window into the status, management and conservation of species?
Source: Conserv Physiol. 2014 Jun 27;2(1):cou023. doi: 10.1093/conphys/cou023 (PMC4732472; doi:10.1093/conphys/cou023)
Supplement: Supplementary Data [file supp_cou023_cou023supp.doc]

**Electronic Supplementary Material for *Measures of Physiological Stress: A Transparent or Opaque Window into the Status, Management, and Conservation of Species?* By Ben Dantzer, Quinn E. Fletcher, Rudy Boonstra, and Michael J. Sheriff in *Conservation Physiology***

**Electronic Supplementary Material for Meta-Analysis**

We attempted to locate all studies that have examined the effect of human disturbance on glucocorticoid levels. We searched in *Web of Science* for the following terms: “conservation” and (“glucocorticoid*” or “cortisol” or “corticosterone”). For all relevant articles, we also searched their references, and searched the articles that cited them (i.e., backward and forward searches). The following types of studies were excluded from the meta-analysis: 1) studies that focused on captivity as a stressor (Carder and Semple, 2008; Setchell et al., 2008; Vick et al., 2012); 2) studies that focused on stress imposed by human hunting (Roy and Woolf, 2001); 3) glucocorticoid responses to pollutants (Hopkins et al., 1997; Verboven et al., 2010); 4) studies that quantified human disturbance as a continuous variable (Millspaugh et al., 2001; Tempel and Gutiérrez, 2004; Maréchal et al., 2011; Bókony et al., 2012; Aguilar-Melo et al., 2013; Eggermann et al., 2013; Strasser and Heath, 2013); and 5) studies that focused on sub-adults (Suorsa et al., 2003; Müllner et al., 2004; Walker et al., 2005; Partecke et al., 2006; Leshyk et al., 2012; Crino et al., 2013). In studies where data were presented on both sub-adults and adults, we excluded the data from sub-adults and calculated effect sizes only with the data from adults (e.g. Munshi-South et al., 2008; Butler et al., 2013).

Human disturbance was categorized in four ways: 1) habitat modification, 2) noise or disturbance from machines, 3) tourism, and 4) urban habitat. Disturbance resulting from habitat modification was attributed to forestry, agriculture, oil and gas exploration, and mining. Noise or disturbance from machines was attributed to military machines, oil and gas activity, vehicles, chainsaws, snowmobiles and boats. Human disturbance resulting from tourism was attributed to wildlife observations and self-propelled winter sports (e.g., skiing). Finally, human disturbance resulting from urban habitat includes studies where sites within urban areas were compared to sites not in urban areas. The study by Tingvold et al. (2013) was difficult to classify into these four groups. We classified it as noise or disturbance from machines because there was more traffic in the human disturbed sites; however, the authors also suggest that stress may have resulted from the threat of illegal hunting.

We performed our meta-analysis using J-corrected, Hedges's *g* effect sizes (Hedges, 1981). Hedges's *g* was calculated as the difference between means of the human disturbance and control treatments divided by their pooled standard deviation (Nakagawa and Cuthill, 2007 eqns. 1 & 2). The J-value is a correction factor that is used to correct for biases imposed by small sample sizes (Gurevitch and Hedges, 2001; eqn. 18.3). Means, standard deviations (or standard errors), and sample sizes of human disturbance and control treatments were extracted either from the text or from figures using Plot Digitizer Ver. 2.6.3 (<http://plotdigitizer.sourceforge.net/>). The calculation of an effect size from means and standard deviations assumes that the data in the treatments are normally distributed (Gurevitch and Hedges, 2001). We note in Table 1 which studies presented normally distributed data, and thus, met the assumptions for the calculation of an effect size. For some studies, we calculated Hedges's *g* by converting *t-* and *F-*values (eqns. 10 and 3; Nakagawa and Cuthill, 2007). Information relating to where or how we obtained the data from the articles is found in Table 1.

When all necessary data could not be extracted, we contacted the corresponding authors (and/or the lead authors) to request these data. Eight studies were removed from the analysis because the authors did not include the measures required for the meta-analysis and they did not respond to our multiple requests for data. These studies included five examining mammals (Gobush et al., 2008; Ahlering et al., 2013: *Loxodonta africana*; Barja et al., 2007: *Martes martes*; Piñeiro et al., 2012: *Felis silvestris*; von der Ohe et al., 2004: *Ursus arctos horribilis*), two examining birds (Thiel et al., 2008; Thiel et al., 2011: *Tetrao urogallus*), and one examining fish (Blevins et al., 2014: *Semotilus atromaculatus*).

For a subset of articles included in the meta-analysis, we extracted more than one effect size. This occurred for four possible reasons. First, the effect of human disturbance on glucocorticoids was examined for multiple species in the article (Creel et al., 2002; Fokidis et al., 2009; Butler et al., 2013). Second, there was a significant human disturbance by sex interaction on glucocorticoids; thus, we calculated separate effect sizes for males and females (Wasser et al., 1997; Newcomb Homan et al., 2003; Bonier et al., 2007; Hayward et al., 2011; Suárez-Domínguez et al., 2011; Bourbonnais et al., 2013). Third, there was a significant human disturbance by season interaction on glucocorticoids; thus, we calculated separate effect sizes for distinct seasons (Zhang et al., 2011). Fourth, multiple types of human disturbance were examined in the study; thus, we calculated separate effect sizes for each type of disturbance (Lucas et al., 2006).

**Table S1.** All studies that we located that have examined the effect of human disturbance on glucocorticoid levels t. We classified four types of human disturbances: habitat modification, noise or disturbance from machines, tourism, and urban habitat. J-corrected effect sizes (Hedges's *g*) were calculated either using t-values (Nakagawa and Cuthill, 2007) or the means (x), sample sizes (*n*), and standard deviations (sd) of glucocorticoid levels in response to human disturbance and the control. The column “Data Loc.” details where or how we obtained the data for the articles. The column “Normality (y/n)” indicates whether or not the data used to calculate the means and standard deviations were normally distributed. The column “Raw Data (y/n)” indicates whether effect sizes were calculated based on raw data, or whether effect sizes were calculated using partial residuals. “GC sample” describes how glucocorticoid samples were collected. The following units are valid for all effect sizes based on raw data: plasma - ng/mL plasma (baseline samples); fecal – ng/g dry feces, except for Fecal* - ng/g wet feces; fur – pg/mg fur; feather – pg/mm feather; urine - µL cortisol/dL Creatinine. The column “Notes” indicates information regarding related to how the effects of sex and season examined within each study. Labels of “males only” or “females only” marked with a super-script “†” were from studies where there was a significant human disturbance by sex interaction on glucocorticoids, and thus, male- and female-specific effect sizes were calculated.

*See table enclosed*

1. Hopkins and DuRant 2011 , 2. Newcomb Homan et al. 2003, 3. Leshyk et al. 2013, 4. Lucas et al. 2006, 5. Bourbonnais et al. 2013, 6. Jaimez et al. 2012, 7. Martínez-Mota et al. 2007, 8. Navarro-Castilla et al. 2014, 9. Suárez-Domínguez et al. 2011, 10. Barron et al. 2012, 11. Blickley et al. 2012, 12. Butler et al. 2013, 13. Dietz et al. 2013, 14. Hayward et al. 2011, 15. Morgan et al. 2012, 16. Tempel and Gutiérrez 2004, 17. Wasser et al. 1997, 18. Allen et al. 2011, 19. Creel et al. 2002 20. Garcia Pereira et al. 2006, 21. Munshi-South et al. 2008, 22. Rolland et al. 2012, 23. Spercoski et al. 2012, 24. Tingvold et al. 2013, 25. Van Meter et al. 2009, 26. Arlettaz et al. 2007, 27. Barbosa et al. 2013 28. Ellenberg et al. 2007, 29. Fowler 1999, 30. Villanueva et al. 2012, 31. Walker et al. 2005, 32. Rangel-Negrín et al. 2009, 33. Behie et al. 2010, 34. Muehlenbein et al. 2012, 35. Rehnus et al. 2014, 36. Zwijacz-Kozica et al. 2013, 37. French et al. 2010, 38. Romero and Wikelski 2002, 39. Bonier et al. 2007, 40. Davies et al. 2013, 41. Fokidis et al. 2009, 42. Schoech et al. 2007, 43. Zhang et al. 2011, 44. Dowle et al. 2013, 45. French et al. 2008, 46. Lucas and French 2012.

**References**

Aguilar-Melo AR, Andresen E, Cristóbal-Azkarate J, Arroyo-Rodríguez V, Chavira R, Schondube J, Serio-Silva JC, Cuarón AD (2013) Behavioral and physiological responses to subgroup size and number of people in howler monkeys inhabiting a forest fragment used for nature-based tourism. Am J Primatol 75:1108-1116.

Ahlering MA, Maldonado JE, Eggert LS, Fleischer RC, Western D, Brown JL (2013) Conservation outside protected areas and the effect of human-dominated landscapes on stress hormones in savannah elephants. Conserv Biol 27:569-575.

Allen LC, Turmelle AS, Widmaier EP, Hristov NI, McCracken GF, Kunz TH (2011) Variation in physiological stress between bridge-and cave-roosting Brazilian free-tailed bats. Conserv Biol 25:374-381.

Arlettaz R, Patthey P, Baltic M, Leu T, Schaub M, Palme R, Jenni-Eiermann S (2007) Spreading free-riding snow sports represent a novel serious threat for wildlife. Proc R Soc Lond B Biol Sci 274:1219-1224.

Barbosa A, De Mas E, Benzal J, Diaz JI, Motas M, Jerez S, Pertierra L, Benayas J, Justel A, Lauzurica P, Garcia-Pena FJ, Serrano T (2013) Pollution and physiological variability in gentoo penguins at two rookeries with different levels of human visitation. Anarct Sci 25:329-338.

Barja I, Silván G, Rosellini S, Piñeiro A, González-Gil A, Camacho L, Illera JC (2007) Stress physiological responses to tourist pressure in a wild population of European pine marten. J Steroid Biochem 104:136-142.

Barron DG, Brawn JD, Butler LK, Romero LM, Weatherhead PJ (2012) Effects of military activity on breeding birds. J Wildlife Manage 76:911-918.

Behie AM, Pavelka MSM, Chapman CA (2010) Sources of variation in fecal cortisol levels in howler monkeys in Belize. Am J Primatol 72:600-606.

Blevins ZW, Wahl DH, Suski CD (2014) Reach-scale land use drives the stress responses of a resident stream fish. Physiol Biochem Zool 87:113-124.

Blickley JL, Word KR, Krakauer AH, Phillips JL, Sells SN, Taff CC, Wingfield JC, Patricelli GL (2012) Experimental chronic noise is related to elevated fecal corticosteroid metabolites in lekking male Greater Sage-Grouse (*Centrocercus urophasianus*). PLOS ONE 7:e50462.

Bókony V, Seress G, Nagy S, Lendvai ÁZ, Liker A (2012) Multiple indices of body condition reveal no negative effect of urbanization in adult house sparrows. Landscape and Urban Planning 104:75-84.

Bonier F, Martin PR, Sheldon KS, Jensen JP, Foltz SL, Wingfield JC (2007) Sex-specific consequences of life in the city. Behav Ecol 18:121-129.

Bourbonnais ML, Nelson TA, Cattet MRL, Darimont CT, Stenhouse GB (2013) Spatial analysis of factors influencing long-term stress in the grizzly bear (*Ursus arctos*) population of Alberta, Canada. PLOS ONE 8:e83768.

Butler LK, Ries L, Bisson I.-A, Hayden TJ, Wikelski MM, Romero LM (2013) Opposite but analogous effects of road density on songbirds with contrasting habitat preferences. Anim Conserv 16:77-85.

Carder G, Semple S (2008) Visitor effects on anxiety in two captive groups of western lowland gorillas. Applied Animal Behaviour Science 115:211-220.

Creel S, Fox JE, Hardy A, Sands J, Garrott B, Peterson RO (2002) Snowmobile activity and glucocorticoid stress responses in wolves and elk. Conserv Biol 16:809-814.

Crino OL, Johnson EE, Blickley JL, Patricelli GL, Breuner CW (2013) Effects of experimentally elevated traffic noise on nestling white-crowned sparrow stress physiology, immune function and life history. J Exp Biol 216:2055-2062.

Davies S, Rodriguez NS, Sweazea KL, Deviche P (2013) The effect of acute stress and long-term corticosteroid administration on plasma metabolites in an urban and desert songbird. Physiol Biochem Zool 86:47-60.

Dietz MS, Murdock CC, Romero LM, Ozgul A, Foufopoulos J (2013) Distance to a road is associated with reproductive success and physiological stress response in a migratory landbird. The Wilson Journal of Ornithology 125:50-61.

Dowle M, Webster KN, Deane E (2013) Faecal glucocorticoid metabolite concentrations in the free-ranging bandicoots (*Perameles nasuta* and *Isoodon obesulus*) of northern Sydney. Aust Mammal 35:1-7.

Eggermann J, Theuerkauf J, Pirga B, Milanowski A, Gula R (2013) Stress-hormone levels of wolves in relation to breeding season, pack size, human activity, and prey density. Ann Zool Fennici 50:170-175.

Ellenberg U, Setiawan AN, Cree A, Houston DM, Seddon PJ (2007) Elevated hormonal stress response and reduced reproductive output in Yellow-eyed Penguins exposed to unregulated tourism. Gen Comp Endocrinol 152:54-63.

Fokidis HB, Orchinik M, Deviche P (2009) Corticosterone and corticosteroid binding globulin in birds: relation to urbanization in a desert city. Gen Comp Endocrinol 160:259-270.

Fowler GS (1999) Behavioral and hormonal responses of Magellanic penguins (*Spheniscus magellanicus*) to tourism and nest site visitation. Biol Conserv 90:143-149.

French SS, DeNardo DF, Greives TJ, Strand CR, Demas GE (2010) Human disturbance alters endocrine and immune responses in the Galapagos marine iguana (*Amblyrhynchus cristatus*). Horm Behav 58:792-799.

French SS, Fokidis HB, Moore MC (2008) Variation in stress and innate immunity in the tree lizard (*Urosaurus ornatus*) across an urban-rural gradient. J Comp Physiol B 178:997-1005.

Garcia Pereira RJ, Barbanti Duarte JM, Negrão JA (2006) Effects of environmental conditions, human activity, reproduction, antler cycle and grouping on fecal glucocorticoids of free-ranging Pampas deer stags (*Ozotoceros bezoarticus bezoarticus*). Horm Behav 49:114 - 122.

Gobush KS, Mutayoba BM, Wasser SK (2008) Long-term impacts of poaching on relatedness, stress physiology, and reproductive output of adult female African elephants. Conserv Biol 22:1590-1599.

Hayward LS, Bowles AE, Ha JC, Wasser SK (2011) Impacts of acute and long-term vehicle exposure on physiology and reproductive success of the Northern Spotted Owl. Ecosphere 2:art65.

Hedges LV (1981) Distributional theory for Glass’s estimator of effect size and related estimators. Journal of Educational Statistics 6:107–128.

Hopkins WA, Mendonça MT, Congdon JD (1997) Increased circulating levels of testosterone and corticosterone in southern toads, *Bufo terrestris*, exposed to coal combustion waste. Gen Comp Endocrinol 108:237-246.

Jaimez NA, Bribiescas RG, Aronsen GP, Anestis SA, Watts DP (2012) Urinary cortisol levels of gray-cheeked mangabeys are higher in disturbed compared to undisturbed forest areas in Kibale National Park, Uganda. Anim Conserv 15:242-247.

Leshyk R, Nol E, Burke DM, Burness G (2012) Logging affects fledgling sex ratios and baseline corticosterone in a forest songbird. PLOS ONE 7:e33124.

Lucas LD, French SS (2012) Stress-induced tradeoffs in a free-living lizard across a variable landscape: consequences for individuals and populations. PLOS ONE 7:e49895.

Maréchal L, Semple S, Majolo B, Qarro M, Heistermann M, MacLarnon A (2011) Impacts of tourism on anxiety and physiological stress levels in wild male Barbary macaques. Biol Conserv 144:2188-2193.

Martínez-Mota R, Valdespino C, Sánchez-Ramos MA, Serio-Silva JC (2007) Effects of forest fragmentation on the physiological stress response of black howler monkeys. Anim Conserv 10:374-379.

Millspaugh JJ, Woods RJ, Hunt KE, Raedeke KJ, Brundige GC, Washburn BE, Wasser SK (2001) Fecal glucocorticoid assays and the physiological stress response in elk. Wildlife Soc B 29:899-907.

Morgan GM, Wilcoxen TE, Rensel MA, Schoech SJ (2012) Are roads and traffic sources of physiological stress for the Florida Scrub-Jay? Wildlife Res 39:301-310.

Muehlenbein MP, Ancrenaz M, Sakong R, Ambu L, Prall S, Fuller G, Raghanti MA (2012) Ape conservation physiology: fecal glucocorticoid responses in wild *Pongo pygmaeus morio* following human visitation. PLOS ONE 7:e33357.

Müllner A, Eduard Linsenmair K, Wikelski M (2004) Exposure to ecotourism reduces survival and affects stress response in hoatzin chicks (*Opisthocomus hoazin*). Biol Conserv 118:549-558.

Munshi-South J, Tchignoumba L, Brown J, Abbondanza N, Maldonado JE, Henderson A, Alonso A (2008) Physiological indicators of stress in African forest elephants (*Loxodonta africana cyclotis*) in relation to petroleum operations in Gabon, Central Africa. Divers Distrib 14:995-1003.

Navarro-Castilla Á, Barja I, Olea PP, Piñeiro A, Mateo-Tomás P, Silván G, Illera JC (2014) Are degraded habitats from agricultural crops associated with elevated faecal glucocorticoids in a wild population of common vole (*Microtus arvalis*)? Mammal Biol 79:36-43.

Newcomb Homan R, Regosin JV, Rodrigues DM, Reed JM, Windmiller BS, Romero LM (2003) Impacts of varying habitat quality on the physiological stress of spotted salamanders (*Ambystoma maculatum*). Anim Conserv 6:11-18.

von der Ohe CG, Wasser SK, Hunt KE, Servheen C (2004) Factors associated with fecal glucocorticoids in Alaskan brown bears (*Ursus arctos horribilis*). Physiol Biochem Zool 77:313-320.

Partecke J, Schwabl I, Gwinner E (2006) Stress and the city: urbanization and its effects on the stress physiology in European Blackbirds. Ecology 87:1945-1952.

Piñeiro A, Barja I, Silván G, Illera JC (2012) Effects of tourist pressure and reproduction on physiological stress response in wildcats: management implications for species conservation. Wildlife Res 39:532-539.

Rangel-Negrín A, Alfaro JL, Valdez RA, Romano MC, Serio-Silva JC (2009) Stress in Yucatan spider monkeys: effects of environmental conditions on fecal cortisol levels in wild and captive populations. Anim Conserv 12:496-502.

Rehnus M, Wehrle M, Palme R (2014) Mountain hares *Lepus timidus* and tourism: stress events and reactions. J Appl Ecol 51:6-12.

Rolland RM, Parks SE, Hunt KE, Castellote M, Corkeron PJ, Nowacek DP, Wasser SK, Kraus SD (2012) Evidence that ship noise increases stress in right whales. Proc R Soc Lond B Biol Sci 279:2363-2368.

Romero LM, Wikelski M (2002) Exposure to tourism reduces stress-induced corticosterone levels in Galapagos marine iguanas. Biol Conserv 108:371-374.

Roy C, Woolf A (2001) Effects of hunting and hunting-hour extension on Mourning Dove foraging and physiology. J Wildlife Manage 65:808-815.

Schoech SJ, Bowman R, Bridge ES, Boughton RK (2007) Baseline and acute levels of corticosterone in Florida Scrub-Jays (*Aphelocoma coerulescens*): effects of food supplementation, suburban habitat, and year. Gen Comp Endocrinol 154:150-160.

Setchell JM, Smith T, Wickings EJ, Knapp LA (2008) Factors affecting fecal glucocorticoid levels in semi-free-ranging female mandrills (*Mandrillus sphinx*). Am J Primatol 70:1023-1032.

Spercoski KM, Morais RN, Morato RG, de Paula RC, Azevedo FC, May-Júnior JA, Santos JP, Reghelin AL, Wildt DE, Songsasen N (2012) Adrenal activity in maned wolves is higher on farmlands and park boundaries than within protected areas. Gen Comp Endocrinol 179:232-240.

Strasser EH, Heath JA (2013) Reproductive failure of a human-tolerant species, the American kestrel, is associated with stress and human disturbance. J Appl Ecol 50:912–919.

Suárez-Domínguez EA, Morales-Mávil JE, Chavira R, Boeck L (2011) Effects of habitat perturbation on the daily activity pattern and physiological stress of the spiny tailed iguana (*Ctenosaura acanthura*). Amphibia-Reptilia 32:315-322.

Suorsa P, Huhta E, Nikula A, Nikinmaa M, Jäntti A, Helle H, Hakkarainen H (2003) Forest management is associated with physiological stress in an old-growth forest passerine. Proc R Soc Lond B Biol Sci 270:963-969.

Tempel DJ, Gutiérrez R (2004) Factors related to fecal corticosterone levels in California spotted owls: implications for assessing chronic stress. Conserv Biol 18:538-547.

Thiel D, Jenni-Eiermann S, Braunisch V, Palme R, Jenni L (2008) Ski tourism affects habitat use and evokes a physiological stress response in Capercaillie *Tetrao urogallus*: a new methodological approach. J Appl Ecol 45:845-853.

Thiel D, Jenni-Eiermann S, Palme R, Jenni L (2011) Winter tourism increases stress hormone levels in the Capercaillie *Tetrao urogallus*. Ibis 153:122-133.

Tingvold HG, Fyumagwa R, Bech C, Baardsen LF, Rosenlund H, Røskaft E (2013) Determining adrenocortical activity as a measure of stress in African elephants (*Loxodonta africana*) in relation to human activities in Serengeti ecosystem. Afr J Ecol 51:580-589.

Van Meter PE, French JA, Dloniak SM, Watts HE, Kolowski JM, Holekamp KE (2009) Fecal glucocorticoids reflect socio-ecological and anthropogenic stressors in the lives of wild spotted hyenas. Horm Behav 55:329-337.

Verboven N, Verreault J, Letcher RJ, Gabrielsen GW, Evans NP (2010) Adrenocortical function of Arctic-breeding Glaucous Gulls in relation to persistent organic pollutants. General and Comparative Endocrinology 166:25 - 32.

Vick MM, Wildt DE, Turner JB, Palme R, Wolfe BA, Pukazhenthi BS (2012) Glucocorticoid response to changes in enclosure size and human proximity in the Persian onager (*Equus hemionus onager*). Stress 15:52-61.

Villanueva C, Walker BG, Bertellotti M (2012) A matter of history: effects of tourism on physiology, behaviour and breeding parameters in Magellanic Penguins (*Spheniscus magellanicus*) at two colonies in Argentina. J Ornithol 153:219-228.

Walker BG, Dee Boersma P, Wingfield JC (2005) Physiological and behavioral differences in Magellanic penguin chicks in undisturbed and tourist-visited locations of a colony. Conserv Biol 19:1571-1577.

Wasser SK, Bevis K, King G, Hanson E (1997) Noninvasive physiological measures of disturbance in the Northern Spotted Owl. Conserv Biol 11:1019-1022.

Zhang S, Lei F, Liu S, Li D, Chen C, Wang P (2011) Variation in baseline corticosterone levels of Tree Sparrow (*Passer montanus*) populations along an urban gradient in Beijing, China. J Ornithol 152:801-806.

Zwijacz-Kozica T, Selva N, Barja I, Silván G, Martínez-Fernández L, Illera JC, Jodłowski M (2013) Concentration of fecal cortisol metabolites in chamois in relation to tourist pressure in Tatra National Park (South Poland). Acta Theriol 58:215-222.
